# Competition between two Usutu virus isolates in cell culture and in the common house mosquito *Culex pipiens*

**DOI:** 10.3389/fmicb.2023.1195621

**Published:** 2023-05-24

**Authors:** Joyce W. M. van Bree, Charlotte Linthout, Teije van Dijk, Sandra R. Abbo, Jelke J. Fros, Constantianus J. M. Koenraadt, Gorben P. Pijlman, Haidong Wang

**Affiliations:** ^1^Laboratory of Virology, Wageningen University and Research, Wageningen, Netherlands; ^2^Laboratory of Entomology, Wageningen University and Research, Wageningen, Netherlands

**Keywords:** Usutu virus, co-infection, competition, vector competence, isolates, mosquito vector, *Culex pipiens*

## Abstract

Usutu virus (USUV) is a mosquito-borne flavivirus of African origin. Over the past decades, USUV has spread through Europe causing mass die-offs among multiple bird species. The natural transmission cycle of USUV involves *Culex spp*. mosquitoes as vectors and birds as amplifying hosts. Next to birds and mosquitoes, USUV has also been isolated from multiple mammalian species, including humans, which are considered dead-end hosts. USUV isolates are phylogenetically classified into an African and European branch, subdivided into eight genetic lineages (Africa 1, 2, and 3 and Europe 1, 2, 3, 4, and 5 lineages). Currently, multiple African and European lineages are co-circulating in Europe. Despite increased knowledge of the epidemiology and pathogenicity of the different lineages, the effects of co-infection and transmission efficacy of the co-circulating USUV strains remain unclear. In this study, we report a comparative study between two USUV isolates as follows: a Dutch isolate (USUV-NL, Africa lineage 3) and an Italian isolate (USUV-IT, Europe lineage 2). Upon co-infection, USUV-NL was consistently outcompeted by USUV-IT in mosquito, mammalian, and avian cell lines. In mosquito cells, the fitness advantage of USUV-IT was most prominently observed in comparison to the mammalian or avian cell lines. When *Culex pipiens* mosquitoes were orally infected with the different isolates, no overall differences in vector competence for USUV-IT and USUV-NL were observed. However, during the *in vivo* co-infection assay, it was observed that USUV-NL infectivity and transmission were negatively affected by USUV-IT but not *vice versa*.

## Graphical Abstract

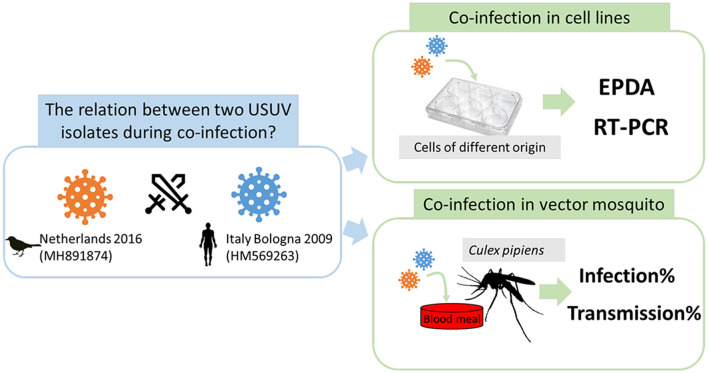

## 1. Introduction

The sudden mass mortality in Eurasian blackbirds in Austria in 2001 marked the arrival of the Usutu virus (USUV), which is a mosquito-borne flavivirus, in the European continent (Weissenböck et al., 2002). Since the outbreak in 2001, the relatively harmless image of USUV to birds has shifted toward a more severe one, as USUV rapidly expanded its geographic distribution in Europe, causing re-emerging outbreaks in birds, particularly blackbirds (*Turdus merula*) and great gray owls (*Strix nebulosa*). USUV disease is characterized by severe central nervous system disorders and high mortality rates (Vilibic-Cavlek et al., 2020). In Europe, USUV is mainly transmitted between the common house mosquito (*Culex pipiens*) and blackbirds (*Turdus merula*) (Chvala et al., 2007; Fros et al., 2015a; Weidinger et al., 2020). In addition to mosquitoes and birds, USUV antibodies and USUV RNA are sporadically detected in other mammals such as horses, dogs, bats, and humans (Engel et al., 2016; Clé et al., 2019). Spillover to humans can happen through incidental mosquito bites. While before its emergence in Europe, only two mild cases of USUV infection in humans had been confirmed (Nikolay et al., 2011), the increase in enzootic events in Europe was accompanied by an increase in human infections along with severe neuroinvasive diseases in immunocompromised patients (Cavrini et al., 2009; Pecorari et al., 2009; Santini et al., 2015; Clé et al., 2019).

Usutu virus (USUV) was first discovered in South Africa in 1959 (Nikolay et al., 2011). To date, many RNA sequences of USUV isolates from a diversity of host species have been documented. Genome sequencing revealed that the overall nucleotide (nt) identity among these isolates is above 94%, except for the prototype central African strain ArB1803, which has an overall 80% nt similarity to other USUV strains (Engel et al., 2016). Phylogenetic analyses based on the USUV envelope (E) and the non-structural (NS) protein coding genes revealed eight distinct lineages of USUV which are phylogeographically clustered into the African (Africa 1, 2 and 3) and European (Europe 1, 2, 3, 4 and 5) lineages (Supplementary Figure S1) (Cadar et al., 2017). Furthermore, these studies suggest that USUV has been introduced in Europe multiple times prior to the Austrian outbreak in 2001, resulting in the establishment of diverse genetic lineages in different regions of Europe (Engel et al., 2016; Cadar et al., 2017). Even though genetic differences between the lineages have been well described, it remains largely unknown if and how these distinct genotypes contribute to differences in viral pathogenicity and transmission dynamics.

This is particularly relevant considering that USUV is closely related to another encephalitic flavivirus, West Nile virus (WNV), which has undergone significant changes in the pathogenicity of specific lineages. WNV lineage 1 has historically been the most pathogenic, whereas WNV lineage 2 and strain Kunjin were considered being of low pathogenicity. However, as of 2010, lineage 2 WNV emerged in Europe and now causes annual outbreaks of encephalitic disease (Botha et al., 2008; Papa et al., 2011; Pérez-Ramírez et al., 2017). Similarly, outbreaks of the pathogenic WNV strain Kunjin in horses have been recorded recently (Van Den Hurk et al., 2014; Prow et al., 2016; Musso et al., 2018).

In Europe, the USUV Europe 2 lineage is most frequently detected in birds, mosquitoes, and humans (Engel et al., 2016). Moreover, the Europe 2 lineage is also more often associated with neurological diseases in humans than the other isolates (Engel et al., 2016; Clé et al., 2020). Differences in pathogenicity were also observed in murine models. In Swiss mice, great differences in mortality rate and time-to-death were observed among the different USUV lineages. Specifically, the Europe 2 lineage exhibited the most virulent phenotype with a 100% mortality rate at 10 days post infection, followed by Europe 5 lineage (85%), Africa 3 lineage (80%), Europe 1 lineage (56%), Europe 3 lineage (54%), and Africa 2 lineage (53%) at 20 days post infection. The mean time-to-death rate was also highest for USUV Europe 2 lineage together with Africa 2 lineage (8–9 days post infection), while for USUV Africa 3 lineage, even a delayed time-to-death was observed (16 days post infection) (Clé et al., 2021). *Ifnar*1^−/−^ mice inoculated with an isolate from the Netherlands (USUV Africa 3 lineage, AS201600034, Netherlands, 2016) had significantly lower levels of viremia and a much lower mortality rate (12%) than infections with various other isolates belonging to European and African lineages (100%) (Kuchinsky et al., 2020). These studies provide an experimental basis to monitor the pathogenesis of different USUV lineages.

In addition to investigating pathogenicity, vector competence, i.e., the intrinsic capacity of a mosquito species to transmit a specific virus, lineage, or isolate of that virus, is a crucial factor that determines USUV outbreak potential. However, comparative analyses on differences in transmission by vector mosquitoes between the various USUV lineages are still lacking. Previously, we reported that USUV transmission efficiency by *Culex pipiens* mosquitoes was negatively affected upon co-infection with WNV (Wang et al., 2020). Co-infection may occur in nature, especially in areas where multiple lineages co-circulate. Whether co-circulating USUV lineages also interact with each other and whether these interactions result in changes in transmission dynamics remain unknown.

In the current study, we compared the replication dynamics and interactions between two USUV isolates: (1) a Dutch isolate (2016) belonging to the African lineage 3 (hereafter, referred to as USUV-NL) and (2) an Italian isolate belonging to the European lineage 2 (hereafter, referred to USUV-IT). Viral fitness was assessed in different vertebrate and mosquito cell lines and their natural mosquito vector *Culex pipiens*. Our results indicate competition between the two isolates during co-infection, although co-transmission of both lineages by *Culex pipiens* mosquitoes is still possible.

## 2. Materials and methods

### 2.1. Viruses and cells

Passage 6 virus stock of USUV the Netherlands 2016 (GenBank accession no. MH891847.1; EVAg Ref-SKU 011V-02153; obtained from Erasmus Medical Center, Rotterdam, the Netherlands) and passage 2 virus stock of USUV Bologna 2009 (GenBank accession no. HM569263.1) (Fros et al., 2015a) were grown on Vero E6 cells (ATCC CRL-1586).

African green monkey kidney Vero E6 cells and chicken fibroblast DF-1 cells were cultured in Dulbecco's Modified Eagle Medium (DMEM; Gibco, Carlsbad, CA, USA), with 10% fetal bovine serum (FBS; Gibco), penicillin (100 U/ml; Sigma–Aldrich, Saint Louis, MO, USA), and streptomycin (100 μg/ml; Sigma–Aldrich) (P/S) at 37°C with 5% CO_2_. Before virus infections, Vero and DF-1 were seeded in HEPES-buffered DMEM medium (Gibco) and supplemented with 10% FBS and P/S. When Vero cells were incubated with mosquito body lysate or saliva, the HEPES-buffered DMEM medium was additionally supplemented with gentamycin (50 μg/ml; Gibco) and fungizone (2.5 μg/ml of amphotericin B and 2.1 μg/ml of sodium deoxycholate; Gibco), which, hereafter, will be referred to as DMEM HEPES complete.

Mosquito cell lines *Aedes albopictus* C6/36 (ATCC CRL-1660), U4.4 (Singh, 1967), U4.4 Argonaut 2 k/o (Ago 2 k/o) (Besson et al., 2022), and *Culex tarsalis* Chao Ball (C.B) cells (provided by Dr. Roy Hall, University of Queensland, Australia) (Chao and Ball, 1976) were cultured in Leibovitz's medium (Invitrogen) and supplemented with 10% FBS, 2% tryptose phosphate (Invitrogen), and 1% non-essential amino acids. All mosquito cells were passaged two times a week and kept at 27°C.

### 2.2. End-point dilution assay

Viral titers were determined by end-point dilution assays (EPDAs) on Vero cells and expressed as 50% tissue culture infectious dose per milliliter (TCID_50_/ml). In total, 10-fold serial dilutions of virus stocks and experimental samples were prepared in HEPES-buffered DMEM medium, supplemented with 10% FBS and P/S, and mixed 1:1 with 2.8 x 10^5^/mL Vero cells. Each virus dilution and cell mixture was plated onto 6-fold microtiter plates (Nunc, Sigma–Aldrich). Cells were checked for cytopathic effects (CPE) at 3 and 5 days post infection (dpi), and viral titers were determined using the Reed–Muench method.

### 2.3. Mosquito rearing

A *Culex pipiens* biotype *pipiens* (*Cx. pipiens*) colony from the Netherlands was maintained at 23°C, with a 16:8 light:dark cycle and relative humidity of 60% as described previously (Möhlmann et al., 2018).

### 2.4. Virus infection in cells

For the growth curve analysis, vertebrate Vero and DF-1 cells, mosquito C6/36, C.B, and U4.4 cells were infected with USUV-NL or USUV-IT at a multiplicity of infection (MOI) of 0.1. Supernatants were harvested at 0, 1, 2, 3, 4, and 7 days post infection (dpi), and titers were determined by EPDAs on Vero cells. Upon co-infection, Vero, DF-1, C6/36, C.B, and U4.4 cells were infected with USUV-NL and USUV-IT simultaneously using an MOI of 0.1 or 5 in different combinations. After inoculation, vertebrate cells were incubated at 37°C, and mosquito cells were incubated at 28°C for 2 h. After incubation, the cell culture supernatant was removed, and cells were washed two times with PBS. Fresh cell culture medium was added, and cells were incubated for another 3 days before RNA extraction.

### 2.5. Infectious blood meal

Adults of *Culex pipiens* (7–14 days old) were starved overnight prior to the infectious blood meal. Mosquitoes were then fed with human whole blood (Sanquin Blood Supply Foundation, Nijmegen, the Netherlands) containing 10^7^ TCID_50_/ml of USUV in a dark room for 1 h using a Hemotek PS5 feeder (Discovery Workshops, Lancashire, United Kingdom). After the blood meal, mosquitoes were immobilized using 100% CO_2_, and only the fully engorged females were selected, three of which were immediately stored at −80°C in SafeSeal microtubes (Sarstedt, Nümbrecht, Germany) containing 0.5 mm zirconium oxide beads (Next Advance, Averill Park, NY, USA) to measure the virus uptake after engorgement. The remaining females were incubated at 28°C for 14 days and supplied with a 6% glucose solution. After incubation, 112, 115, 72, and 86 mosquitoes survived who fed on USUV-NL, USUV-IT, NL:IT=1:5, and NL:IT=5:1, respectively.

### 2.6. Salivation assay

Mosquitoes were immobilized using 100% CO_2_, and their legs and wings were removed. The proboscis was inserted into a 200 μl pipet tip containing 5 μl of a 1:1 mixture of FBS and 50% sugar in autoclaved tap water. The mosquitoes were allowed to salivate for 45 min. After salivation, bodies were collected in SafeSeal microtubes, and the salivation mixtures were collected and transferred to a 55 μl DMEM HEPES complete medium. Both the bodies and saliva samples were stored at −80°C.

### 2.7. Infectivity assay

Frozen mosquito body samples were homogenized in a Bullet Blender Storm (Next Advance), according to a previously reported protocol (Fros et al., 2015b). In total, 30 μl of the body tissue homogenate or mosquito saliva was added to one well of a 96-well plate containing Vero cells at 80% confluency. After incubation for 2 h at 37°C, the cell medium was replaced with fresh DMEM HEPES complete medium, and the cells were kept at 37°C for another 6 days. Positive viral infection was determined by checking for CPE at both 3 dpi and 6 dpi for each well. The infection rate and transmission efficiency were expressed as the percentage of virus-positive mosquito bodies or saliva over the total number of mosquitoes used for the salivation assay. Viral titers were determined by EPDAs using six and four bodies and saliva samples of the USUV-NL and USUV-IT infected mosquitoes, respectively.

### 2.8. RNA extraction and one-step RT PCR

The total RNA of cells and mosquito body homogenates were isolated using TRIzol reagent (Invitrogen), according to the standard manufacturer's instructions. Viral RNA from mosquito saliva samples was extracted and purified using a Mag-Bind Viral RNA 96 kit (Omega). The yields and the quality of the RNA samples were determined by Nanodrop (Thermo). To determine the presence of the two USUV strains in co-infection samples, a one-step RT-PCR using a single pair of primers followed by enzymatic digestion of the DNA amplicon was performed. From the viral RNA, a 900 bp DNA fragment was synthesized and amplified using the Superscript III One-Step kit with Platinum Taq DNA polymerase (Invitrogen) and primers (Forward: 5'-GGATGTTGGTATGGAATGGAGATA-3'; Reverse: 5'-GTCGATTTGCCTGAAATGGTGT-3'), annealing to the viral NS1 to NS2A genes. Restriction enzyme digestion using *Dra*I (NEB) was used to differentiate between USUV-NL and USUV-IT, as the *Dra*I restriction site is only present in the USUV-NL amplicon. DNA band intensity was quantified using GelAnalyzer 19.1 software. Bands having a pixel count of less than 5% of the total pixel count for that specific sample were considered undigested products and therefore excluded from the analysis.

### 2.9. Statistical analyses

The two-way ANOVA was used to compare the viral titers of both USUV stains in the growth kinetic study, and the Mann–Whitney U-test was used to compare the mean viral titers between two log-transformed data sets from the mosquito experiments. Fisher's exact test was used to compare the infection and transmission efficiencies between both USUV strains. Statistical tests were performed in GraphPad Prism 8 and R (version 4.2).

## 3. Results

### 3.1. USUV-IT replicates to higher peak titers than USUV-NL in mosquito cells

**Figure 1:**
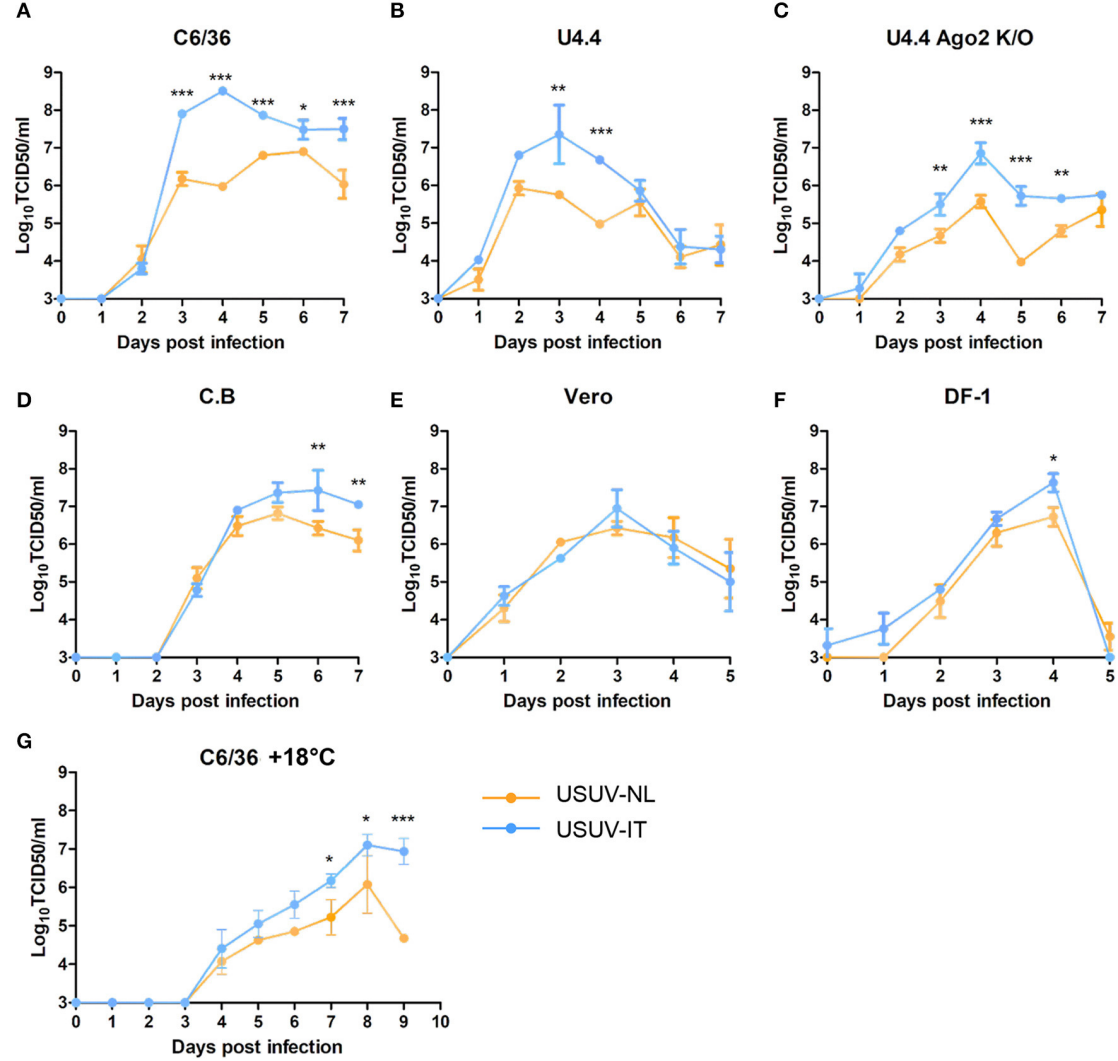
One-step growth curves of USUV-NL and USUV-IT in cells of different origins. **(A, G)** C6/36 (*Aedes albopictus*), **(B)** U4.4 (*Aedes albopictus*), **(C)** U4.4 Ago2 k/o (*Aedes albopictus*), **(D)** C.B (*Culex tarsalis*), **(E)** Vero (*Chlorocebus sabaeus*), and **(F)** DF-1 (*Gallus gallus*). Cells were infected with USUV-NL or USUV-IT at an MOI of 0.1. The supernatant was collected daily, and the virus titer, expressed as TCID_50_/ml, was determined by end-point dilution assay on Vero cells. Error bars represent the standard deviation of independent experiments in triplicate. Blue dots and connection lines indicate USUV-IT (Italy, Bologna, 09 HM569263), and Orange dots and connection lines indicate USUV-NL (the Netherlands, 2016, MH891874). The statistics were carried out by using two-way ANOVA. ^*^, ^**^, and ^***^ represent *p* ≤ 0.05, 0.01, and 0.001, respectively.

To compare the replication dynamics of USUV-NL and USUV-IT, virus replication was examined with one-step growth curves in mammalian, avian, and mosquito cells from various origins. In C6/36 (*Aedes albopictus*), U4.4 and U4.4 Ago2 k/o (*Aedes albopictus*), and C.B (*Culex tarsalis*) cells, USUV-IT replicated to significantly higher peak titers than USUV-NL (Figures 1A–D, *p* ≤ 0.01). C6/36 (Dc2-defective, Figure 1A) and U4.4 Ago2 k/o (Figure 1C) have a compromised antiviral RNAi pathway, but this did not abate the observed replication differences between USUV-NL and USUV-IT.

These experiments were performed at 27°C, the typical incubation temperature of cultured mosquito cells. However, USUV infectivity in *Culex pipiens* mosquitoes is temperature dependent, and the average ambient temperature during the transmission season in the Netherlands is much lower (~18°C) (Fros et al., 2015a,b; Wang et al., 2020). To investigate whether the relative replication dynamics of both isolates is affected by the incubation temperature, C6/36 cells were infected and incubated at 18°C (Figure 1G). For both USUV-NL and USUV-IT, the progression of infection was slower and also peak titers were lower at 18°C than at 27°C. While overall differences in titers between USUV-IT and USUV-NL were smaller at 18°C than at 27°C, USUV-IT still replicated to higher peak titers than USUV-NL (Figure 1G, two-way ANOVA, *p* ≤ 0.05).

The enhanced replication of USUV-IT over USUV-NL observed in mosquito cells was less apparent in mammalian Vero (Figure 1E) and avian DF-1 (Figure 1F) cells. Even though higher peak titers of USUV-IT over USUV-NL were observed in both Vero and DF-1 cells (1.08-fold and 1.25-fold, respectively), a significant difference was only evident at 4 dpi in DF-1 cells (Figure 1F, *p* ≤ 0.05). These results indicate that *in vitro* USUV-IT replicates to higher titers compared with USUV-NL, which is particularly evident in mosquito cells.

### 3.2. USUV-NL is outcompeted by USUV-IT upon co-infection in cells

Next, it was investigated whether the higher viral titers also provided the USUV-IT isolate with a higher competitive fitness compared with USUV-NL. A selection of mosquito and vertebrate cells was inoculated with both viral isolates at an MOI of either 0.1 or 5 and combinations thereof. Culture fluid and cell lysates were collected at 3 dpi, and total RNA was purified. Next, RNA was reverse transcribed, and a PCR amplicon was generated using a single primer pair for both virus isolates. A unique restriction site only present in the USUV-NL PCR amplicon was used to differentiate between USUV-NL and USUV-IT (Supplementary Figure S2).

**Figure 2:**
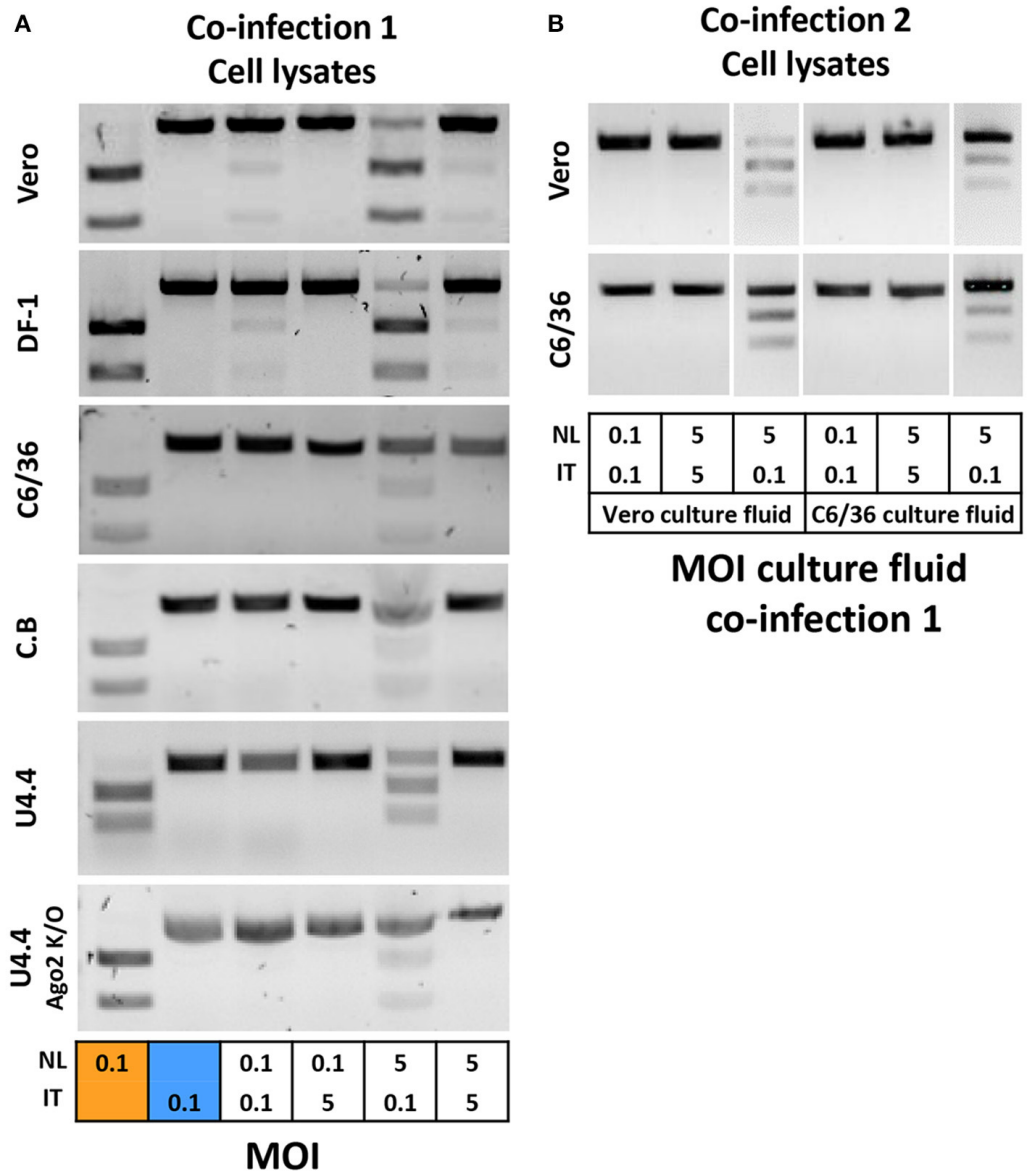
Co-infection of USUV-NL and USUV-IT in cells of different origins. **(A)** Vero, DF-1, C6/36, C. B, U4.4 WT, and U4.4 Ago2 k/o cells were inoculated with both USUV isolates at different MOI combinations of either 0.1 and 1 or 5. At 3 dpi, cell lysates were subjected to RNA extraction followed by RT-PCR and restriction enzyme digestion to determine the presence of both USUV isolates. **(B)** Culture fluid of Vero and C6/36 was inoculated to fresh Vero and C6/36 cells, and virus presence was checked at 3 dpi.

USUV-IT and USUV-NL co-infections using an equivalent MOI of either 0.1 or 5 for both viruses resulted in faint or no detection of USUV-NL in vertebrate cells (Vero and DF-1) or mosquito cells, respectively (Figure 2A). In these co-infected samples, USUV-IT was always clearly detected in both cell lysate and culture medium (Figure 2 and Supplementary Figure S3). When passaging the culture fluid of these co-infected (equal MOI) Vero and C6/36 cells onto fresh Vero or C6/36 cells for another 3 days, bands corresponding to USUV-NL disappeared completely (Figure 2B).

When cells were co-infected using a 50 times lower MOI for USUV-NL compared with USUV-IT (USUV-NL MOI 0.1 and USUV-IT MOI 5), USUV-IT was clearly detected, while USUV-NL was no longer present (Figure 2A). When the inoculum contained a concentration of USUV-NL that was 50 times higher than that of USUV-IT (USUV-NL MOI 5, USUV-IT MOI 0.1), both USUV-NL and USUV-IT were detected at 3 dpi in cell lysates (Figure 2A) and culture fluids (Supplementary Figure S3) of all tested cells. Moreover, the band intensity suggests that the fitness advantage of USUV-IT over USUV-NL was stronger in mosquito cells than vertebrate cells (Figure 2). These results indicate that USUV-IT has a fitness advantage over USUV-NL in all cell types and that this advantage is strongest in mosquito cells compared to the vertebrate cells tested.

### 3.3. USUV competition in *Culex pipiens* mosquitoes

To investigate whether the higher replicative fitness of USUV-IT over USUV-NL is also present in the main mosquito vector of USUV, *Culex pipiens* mosquitoes were subjected to a human blood meal containing either USUV-NL, USUV-IT, or both. Directly after the blood meal, three fully engorged female mosquitoes from each experimental group were homogenized to show that both virus isolates retained similar infectivity while ingested in a blood meal (Supplementary Figure S4). The remaining fully engorged mosquitoes were incubated at 28°C for 14 days before their saliva was collected and bodies were homogenized. All samples were inoculated onto Vero cells to identify infected mosquitoes (bodies) and mosquitoes where the infection had disseminated into the saliva.

**Figure 3:**
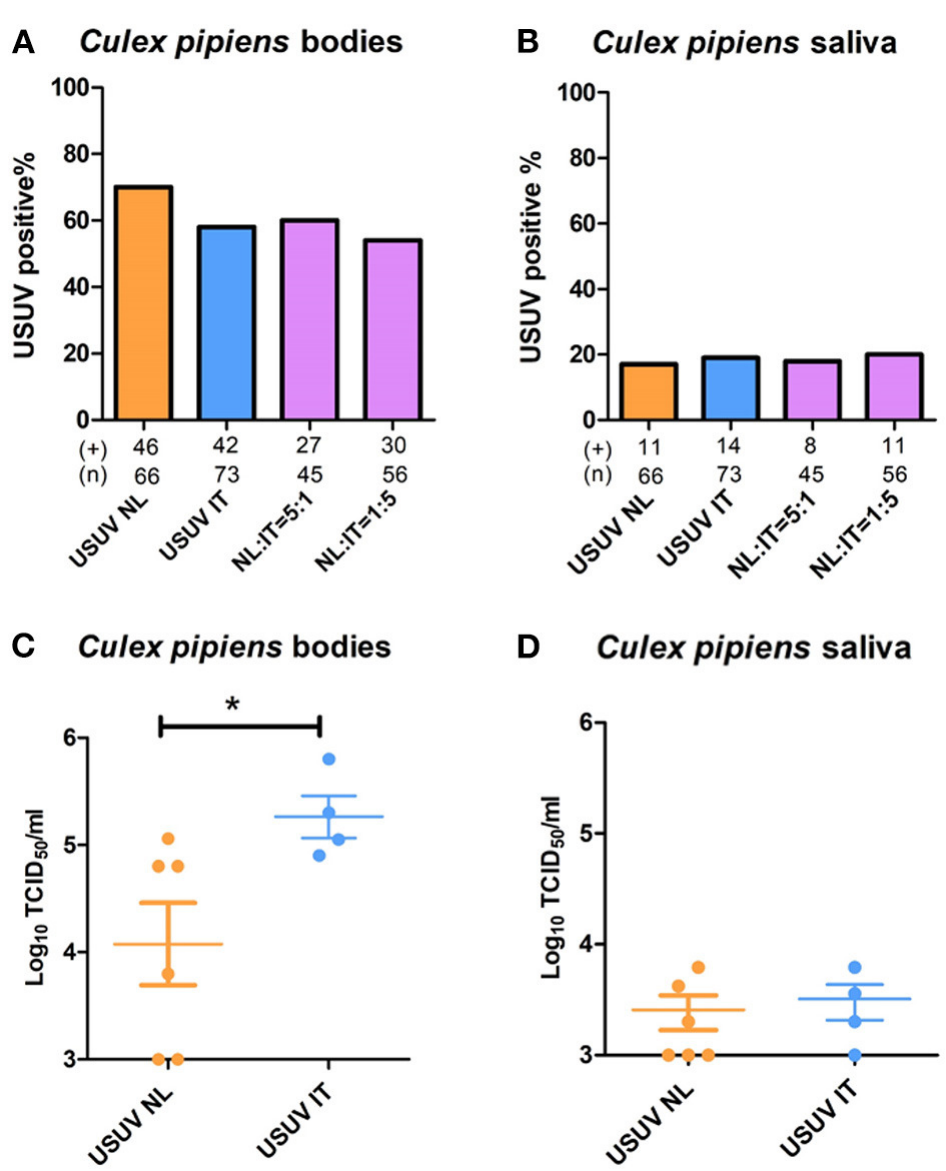
Co-infection of USUV-NL and USUV-IT in *Culex pipiens* mosquitoes. Artificial blood meals containing either USUV-NL and USUV-IT or a mixture of both were provided to 7-day-old mosquitoes. The percentages of virus-positive mosquito body **(A)** and saliva **(B)** were determined 2 weeks after the infectious blood meal. Virus titers in the infected body **(C)** and saliva **(D)** were determined by titrating in Vero cells. Data were collected and pooled from two independent experiments. Fisher's exact test was used to compare the accumulative data; the Mann–Whitney U-test was used to compare the median of the viral titers between the two viral strains; ^*^represents *p* ≤ 0.05.

Ingestion of blood containing solely USUV-NL resulted in 70% positive mosquito bodies compared with 58% in mosquitoes fed with blood containing USUV-IT (Figure 3A). After feeding on blood that contained both USUV-NL and USUV-IT in a 5:1 and 1:5 ratio, 60% and 54% of mosquitoes were infected with USUV, respectively (Figure 3A). These values were not significantly different (Fisher's exact test). Virus dissemination into the mosquito saliva displayed even less variation between virus isolates and mixtures thereof, with 17–20% of engorged female mosquitoes presenting the virus in their saliva (Figure 3B).

Interestingly, while no significant differences in mosquito infection rates were observed, the viral titers in mosquitoes infected solely with USUV-NL (4.1 x 10^4^ TCID_50_/ml) were significantly lower than those infected with USUV-IT ((2.6 x 10^5^ TCID_50_/ml) (Figure 3C); *p* ≤ 0.05, Mann–Whitney U-test). In the saliva, however, no significant difference in viral titers was observed between USUV-NL- and USUV-IT-infected mosquitoes (Figure 3D).

**Figure 4:**
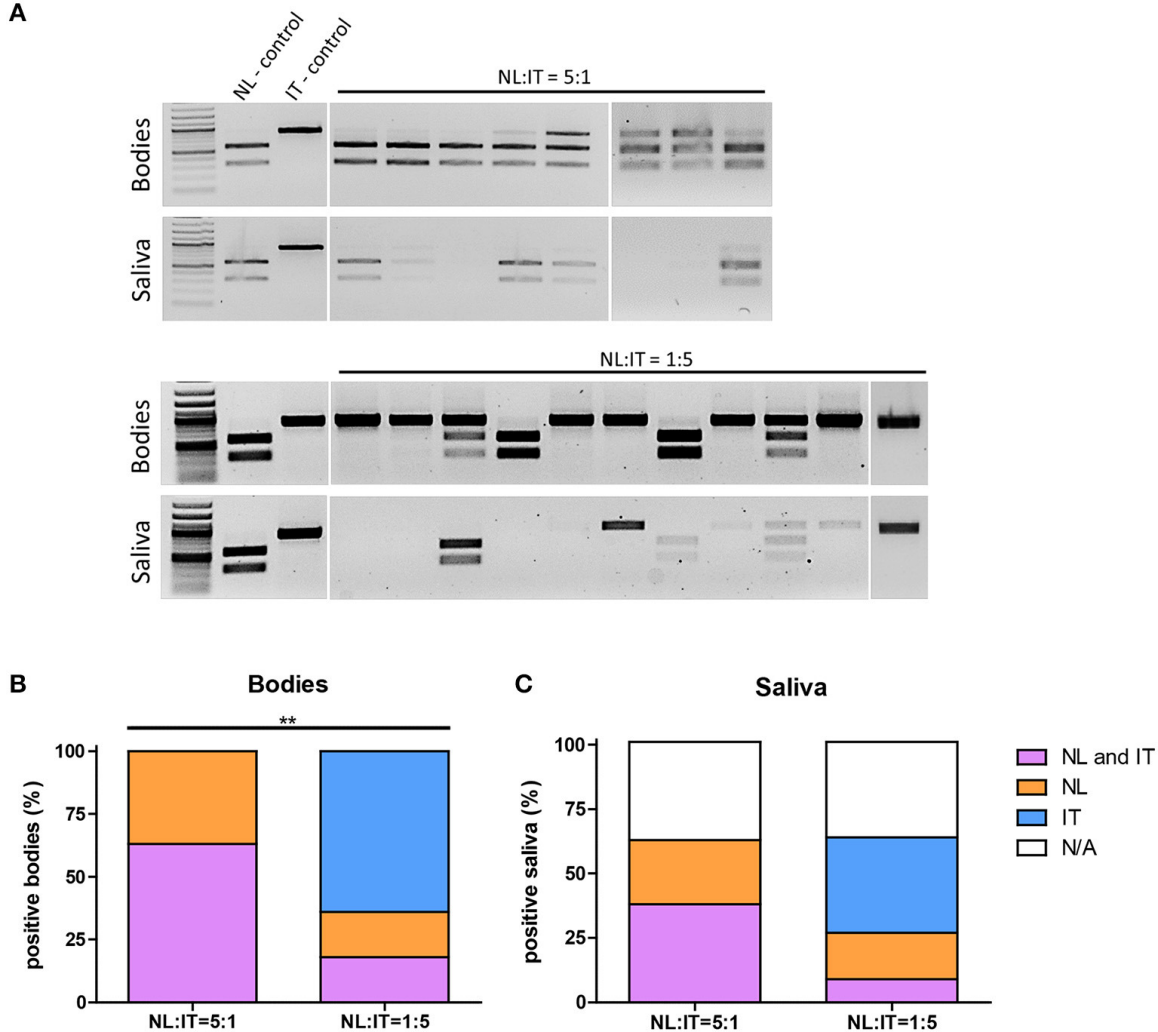
Infectivity of USUV-NL in Cx. pipiens is affected upon co-infection with USUV-IT. Infection rate and transmission efficiency of USUV-NL and USUV-IT calculated following RT-PCR and restriction enzyme digestion **(A)**. Percentage positive body samples **(B)** and saliva samples **(C)** of the total engorged mosquitoes 14 days after blood meal. Data were collected and pooled from two independent experiments. Fisher's exact, one tailed test was used to compare the infection rate and transmission efficiency between the NL:IT = 5:1 and NL:IT = 1:5 co-infected mosquitoes. ^**^USUV-NL positive bodies (includes co-infected bodies) was significantly (*p* ≤ 0.01) lower in the NL:IT = 1:5 than NL:IT = 5:1 co-infected mosquitoes.

Next, RNA was isolated from (co-)infected mosquito bodies and saliva samples followed by RT-PCR. DNA amplicons were digested with the *Dra*I restriction enzyme (Supplementary Figure S2) to distinguish between the Dutch and Italian USUV isolates (Figure 4A). When *Cx. pipiens* female mosquitoes were fed a blood meal containing both isolates with 5-fold less USUV-NL than USUV-IT (1:5), the percentage of mosquito bodies that tested positive for USUV-NL was significantly lower than that of the mosquitoes that were exposed to a blood meal containing both isolates in a ratio of 5:1 (USUV-NL: USUV-IT) (Figure 4B; *p* ≤ 0.001, Fisher's exact test). Interestingly, the percentage of mosquitoes that tested positive for USUV-IT remained almost identical independent of the USUV-NL to USUV-IT ratio and *vice versa* (Figure 4B; percentages). Similarly, the percentage of USUV-NL-positive saliva was lower when mosquitoes were exposed to 5-fold less USUV-NL over USUV-IT (1:5) compared with the mosquitoes that received a blood meal with five times more USUV-NL (5:1) (31% and 60%, respectively; *p* = 0.07, Fisher's exact test) (Figure 4C). The percentage of USUV-IT-positive saliva remained relatively stable independent in which the ratio of both isolates was mixed (40% and 46% in 5:1 and 1:5 ratios, respectively) (Figure 4C). These results suggest that the replicative fitness and transmission efficiency of USUV-IT compared with USUV-NL is greater in *Culex pipiens* mosquitoes. The infectivity of USUV-NL and USUV-NL accumulation in the saliva was suppressed by USUV-IT, whereas the reverse was not observed.

## 4. Discussion

Recent advances in viral genome sequencing have led to substantial expansion of the genomic archive of (re)-emerging arboviruses (Pybus and Rambaut, 2009). This rapid genome data expansion is outpacing the accumulation of our knowledge on virus phenotypes and the consequences thereof on virus–host interactions. By studying different virus isolates, we can obtain a better understanding of replication and transmission phenotypes, which can aid in predicting and monitoring virus spread and pathogenesis. In our study, we examined the interaction between two USUV isolates during co-infection and found that USUV-IT demonstrated superior replicative fitness compared with USUV-NL in mosquito cells and *Cx. pipiens* mosquitoes *in vivo*.

The two USUV isolates studied here phylogenetically belong to the Africa 3 lineage (USUV-NL) and the Europe 2 lineage (USUV-IT) (Supplementary Figure S1). Of all lineages, the USUV Europe 2 lineage is the one being most associated with developing neurological symptoms in infected patients in Europe (Pecorari et al., 2009; Santini et al., 2015; Grottola et al., 2017; Pacenti et al., 2019). In addition, in comparative pathogenesis studies, mice infected with USUV Europe 2 lineage suffered the most from neurological disorders and had the highest mortality rate. Swiss mice infected with USUV Europe 2 lineage had a higher viral burden on their brain compared with USUV Europe 1, 3, and 5 and Africa 2 and 3 lineages-infected mice. In various cultured cells of the central nervous system, USUV Europe 2 lineages replicated to higher peak titers than all the other isolates, and it, furthermore, induced an atypical cytopathic effect characterized by dark detached cell clusters. In all tested cells, the titer of USUV Europe lineage 2 between 2 and 5 days post infection continued to increase over time, suggesting persistent infection, while steeply decreasing for the other lineages (Clé et al., 2021). Similar results were obtained using another USUV lineage 2 isolate, suggesting that these results are lineage rather than isolate dependent (Clé et al., 2021). In another study, mice infected with a Dutch isolate (the Netherlands 2016) belonging to the USUV Africa 3 lineage, which is genetically closely related to USUV-NL, had a much higher survival rate (88% vs. 0%) and significantly lower levels of viremia compared with various other isolates, phylogenetically belonging to different lineages (Kuchinsky et al., 2020).

We found that in cultured mammalian and avian cells, viral replication rates were similar between both USUV isolates except for the peak viral titers, which were higher for USUV-IT. When DF-1 and Vero cells were co-infected with both isolates in our *in vitro* competition assay, the fitness advantage of USUV-IT (Europe 2) over USUV-NL (Africa 3) was readily observed. Strikingly, in all tested mosquito cells, USUV-IT grew to higher (peak) titers than USUV-NL and during co-infection, USUV-NL was only detected when the inoculum contained 50 times more USUV-NL than USUV-IT. Conversely, even when the inoculum contained 50 times more USUV-NL, USUV-IT was present in all other samples indicating USUV-IT rapidly outcompeted USUV-NL. These results indicate that USUV-IT has an advantage over USUV-NL in vertebrates, especially mosquito cells.

Based on our *in vitro* findings, we initially hypothesized that infection of the vector and virus dissemination into the mosquito's saliva would occur more frequently for USUV-IT compared with USUV-NL. However, the infection rate and transmission efficiency of *Cx. pipiens* mosquitoes displayed no significant differences when either USUV-NL or USUV-IT was ingested, although significantly higher USUV-IT than USUV-NL titers were observed in mosquito bodies at 14 dpi. These results suggest that the higher replicative fitness of USUV-IT compared with USUV-NL in mosquito cells either does not contribute to a more effective infection of the mosquito vector or lowers the extrinsic incubation period without affecting the absolute number of infected mosquitoes at 14 dpi. A similar observation was made for a chikungunya virus mutant, where lower infection rates and transmission efficiencies were observed at 3, 6, and 9 dpi but not at 12 dpi compared with the wild-type virus (Merwaiss et al., 2021).

While no differences in vector competence for USUV-IT and USUV-NL were observed in *Cx. pipiens* mosquitoes, the *in vivo* co-infection assay indicated that USUV-NL infectivity and transmission were negatively affected in the presence of USUV-IT but not *vice versa*. Notably, in some saliva samples of mosquitoes infected with five times more USUV-IT than USUV-NL, only USUV-NL was detected. This might suggest that initial infection in the midgut is lower for USUV-IT than USUV-NL, yet once in the cell, USUV-IT replicates at higher rates than USUV-NL. Even though fewer mosquitoes are infected with USUV-IT than USUV-NL, the detection of both viruses in the saliva is equal perhaps suggesting the dissemination rate for USUV-IT is higher than USUV-NL.

Most amino acid substitutions between USUV-NL and USUV-IT are located in the viral envelope (E) and non-structural protein 2A (NS2A) regions (Supplementary Table S1). While it is tempting to speculate that the faster entry and/or reproduction pace of USUV-IT over USUV-NL are the causes for this competition, our ongoing studies aim to attribute differences in the phenotypes between the two isolates to any of these differences in the amino acid sequences.

Our results suggest that co-transmission of both isolates through a single mosquito bite is possible, yet the vector competence of *Culex pipiens* for USUV-NL is negatively affected by USUV-IT but not *vice versa*. The replication and transmission advantage of USUV-IT over USUV-NL in *Cx. pipiens* mosquitoes could mean that USUV-NL eventually may become displaced by USUV-IT in co-circulating areas. Both isolates have been detected in Austria in 2016–2017 during a wave of USUV-associated blackbird deaths (Vilibic-Cavlek et al., 2020). With one exception, the USUV Europe 2 lineage was the dominant lineage detected in Austrian birds (Weidinger et al., 2020). In mosquitoes sampled at the selected sites of bird deaths, only USUV Europe 2 lineage was detected (Camp et al., 2019). Similar to Austria, both isolates have been detected in blackbirds in the Czech Republic, but only USUV Europe 2 lineage was detected in mosquitoes (Hönig et al., 2019). In Germany, both USUV Africa 3 and Europe 2 lineages have been detected in bird populations. In contrast to the Austria scenario, in mosquitoes in Germany, USUV Africa 3 lineage has been detected but not USUV Europe 2 lineage (Sieg et al., 2017; Michel et al., 2019; Vilibic-Cavlek et al., 2020). Co-circulation of different lineages in Europe is thought to be the result of multiple independent introduction events by infected migratory birds from Africa (Engel et al., 2016). The difference in viruses detected in mosquitoes could be the result of the time of introduction. USUV Europe 2 lineage has arisen from USUV Europe 1 lineage. USUV Europe 1 lineage was responsible for the first wave of blackbird deaths in Austria in 2001, indicating that USUV Europe 2 lineage was likely present before the introduction of the USUV Africa 3 lineage in Austria. In Germany, USUV Africa 3 lineage was present before the introduction of the USUV Europe 2 lineage in 2018 (Sieg et al., 2017). It could be that due to the introduction of the USUV Europe 2 lineage, USUV Africa 3 lineage will eventually disappear. However, since additional factors (e.g., immunity in bird populations and co-circulation of WNV in the same area) also play a role in USUV transmission, more information on the transmission dynamics of other co-circulating USUV lineages is necessary to predict the future spread of USUV lineages in Europe.

## 5. Conclusion

In this study, we reported a competitive relation between two USUV isolates belonging to the African (USUV-NL) and European (USUV-IT) lineages, *in vitro* as well as *in vivo* in mosquitoes. We found that USUV-IT has a higher replicative fitness over USUV-NL in mosquito cells and live *Cx. pipiens* mosquitoes. USUV-IT infectivity is less affected upon co-infection than the infectivity of USUV-NL, and a similar trend is also observed for transmission efficiency. Based on our findings, the concurrent transmission of both viral isolates through a single mosquito bite is possible.

## Data availability statement

The original contributions presented in the study are included in the article/Supplementary material, further inquiries can be directed to the corresponding author.

## Author contributions

JvB, CL, TvD, SA, and HW conducted the research. JF, CK, and GP supervised the research. HW and JvB wrote the first draft. JF and GP edited the draft. CK and GP acquired funding. All authors contributed to the article and approved the submitted version.
