# Supplementary material for: Competition between two Usutu virus isolates in cell culture and in the common house mosquito Culex pipiens
Source: Front Microbiol. 2023 May 24;14:1195621. doi: 10.3389/fmicb.2023.1195621 (PMC10244747; doi:10.3389/fmicb.2023.1195621)
Supplement: Supplementary file 1 [file Data_Sheet_1.PDF]

Table S1. Amino acid substitutions between USUV-NL and USUV-IT

Protein	Amino acid	USUV Netherlands 2016	USUV Bologna 2009
C	105	S	G
C	112	L	V
E	345	S	N
E	381	N	D
E	419	V	M
E	472	E	K
E	524	L	S
E	531	T	I
E	595	G	S
E	637	T	S
NS1	830	E	G
NS2	843	Y	H
NS2A	1268	L	F
NS2A	1287	V	A
NS2A	1322	V	I
NS2A	1324	I	T
NS2A	1334	V	A
NS3	1549	F	L
NS3	2059	V	I
NS4B	2287	M	I
NS4B	2460	F	L
NS5	2645	M	I
NS5	2803	S	T
NS5	3060	R	K

Table S2. Infection and transmission efficacy of USUV-NL and USUV-IT in vector mosquito *Culex pipiens*

	Blood meal		
	single infectious	co-infectious (NL:IT=5:1)	co-infecious (NL:IT=1:5)
**NL-positve bodies%**	69.7% (46/66)	12.5% (1/8)	18.2% (2/11)
**IT-positive bodies%**	57.5% (42/73)	0% (0/8)	63.6% (7/11)
**Co-infection bodies%**	/	87.6% (7/8)	18.2% (2/11)
**NL-positve saliva%**	16.7% (11/66)	25% (2/8)	18.2% (2/11)
**IT-positive saliva%**	19.2% (14/73)	0% (0/8)	45.5% (5/11)
**Co-infection saliva%**	/	37.5% (3/8)	9.1% (1/11)
**Undet. saliva%**	/	37.5% (3/8)	27.3% (3/11)

Data were collected and pooled from two independent experiments

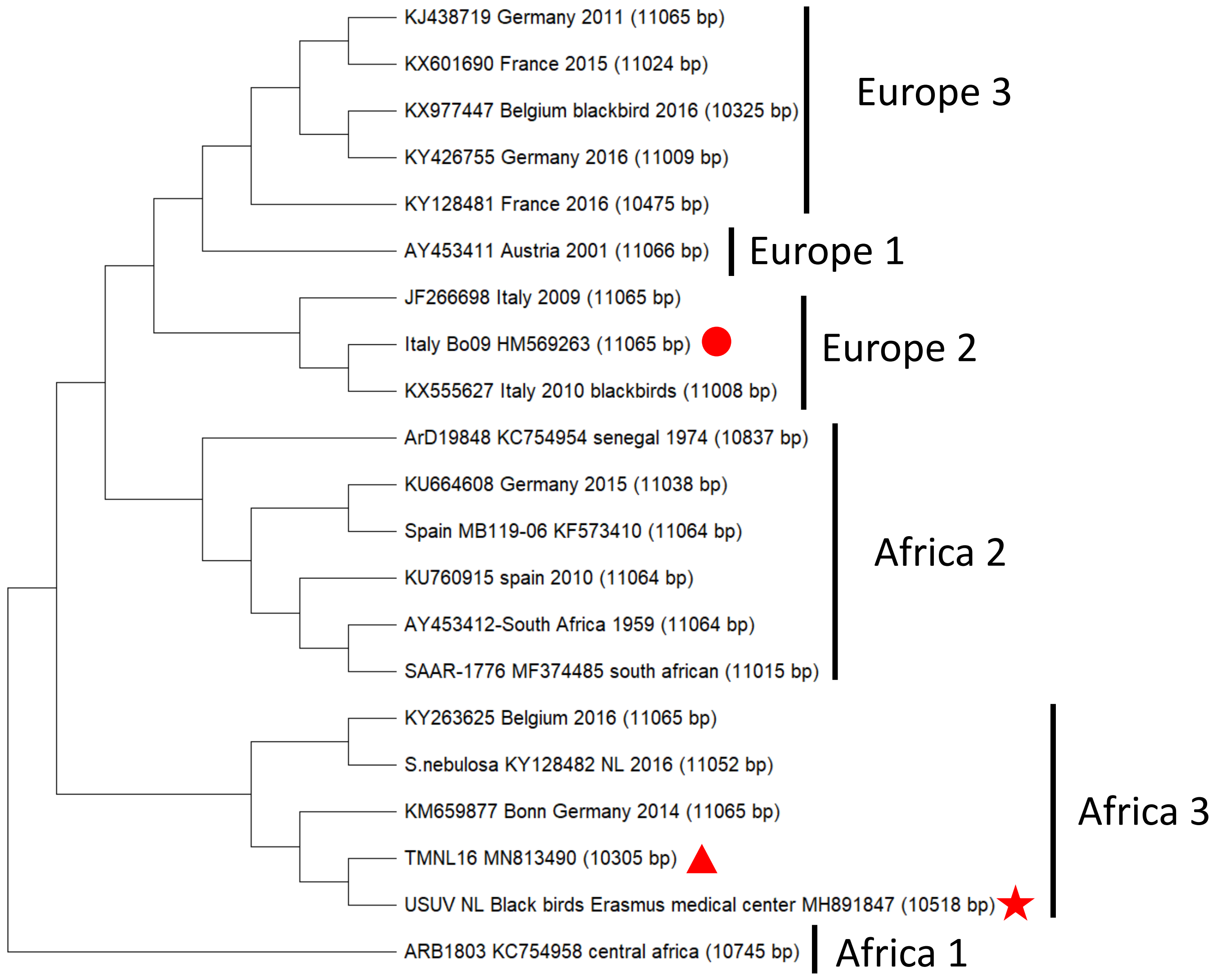

Figure S1. Phylogenetic analysis of the USUV African and European isolates. A maximum like hood phylogenic tree showing the topology was generated using the whole coding region of the USUV isolates. viral sequences were retrieved from the NCBI database. The Red closed circle and star indicate the Italian USUV isolate and Dutch USUV isolate used in the current study, while the red closed triangle represents a Dutch isolate used in a recent study (Kuchinsky SC., et al., 2020 Plos Negl Trop D). The phylogenetic analysis and tree was conducted in MEGA 11.

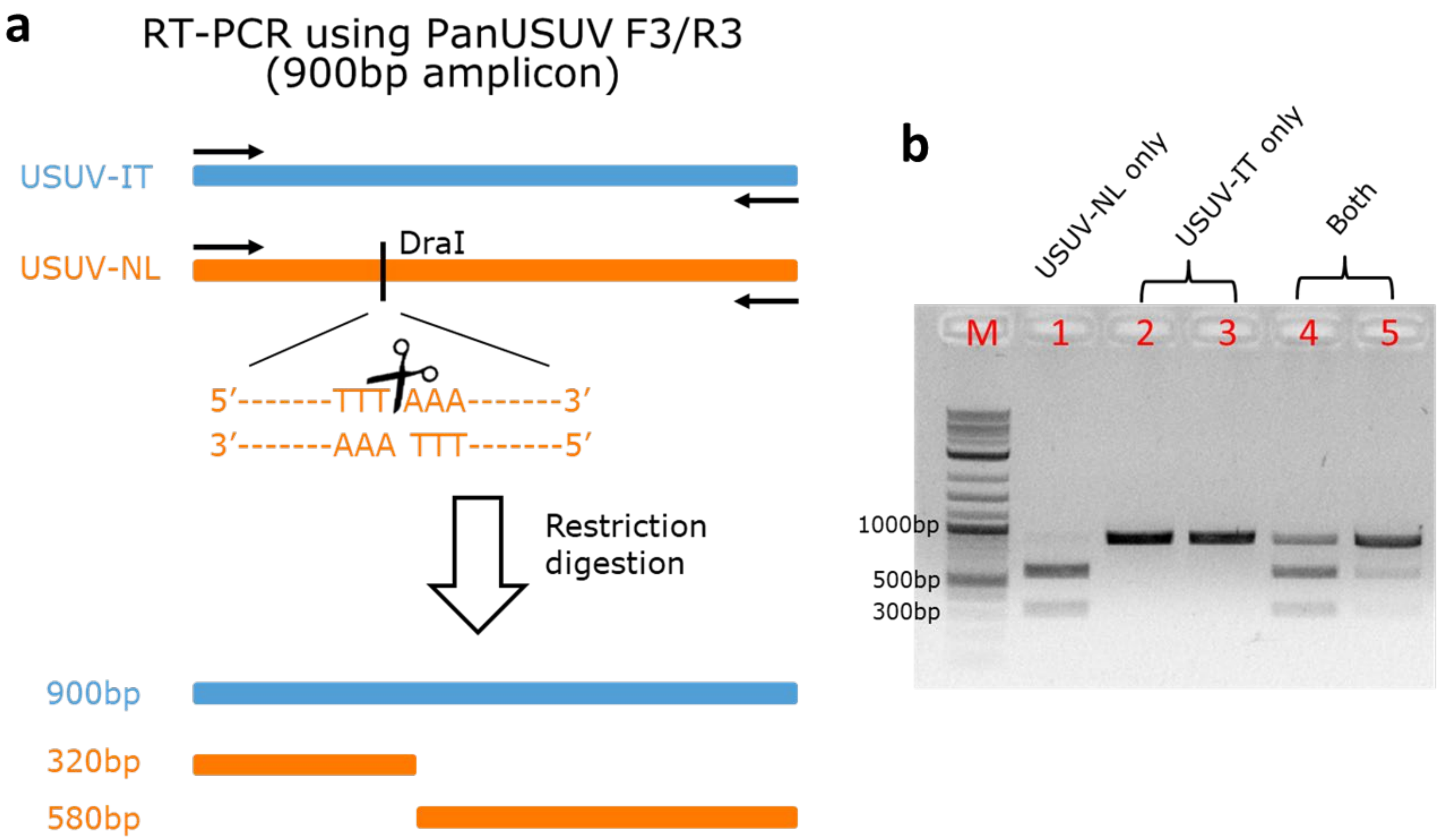

Figure S2. A newly developed RT-PCR with enzyme digestion to identify USUV-NL and USUV-IT in co-infection samples. **(a)** The schematic overview of the primer pair design and restriction treatment. **(b)** Verification of the newly development RT-PCR system in single (lane 1-3) and co-infection (lane 4-5) samples.

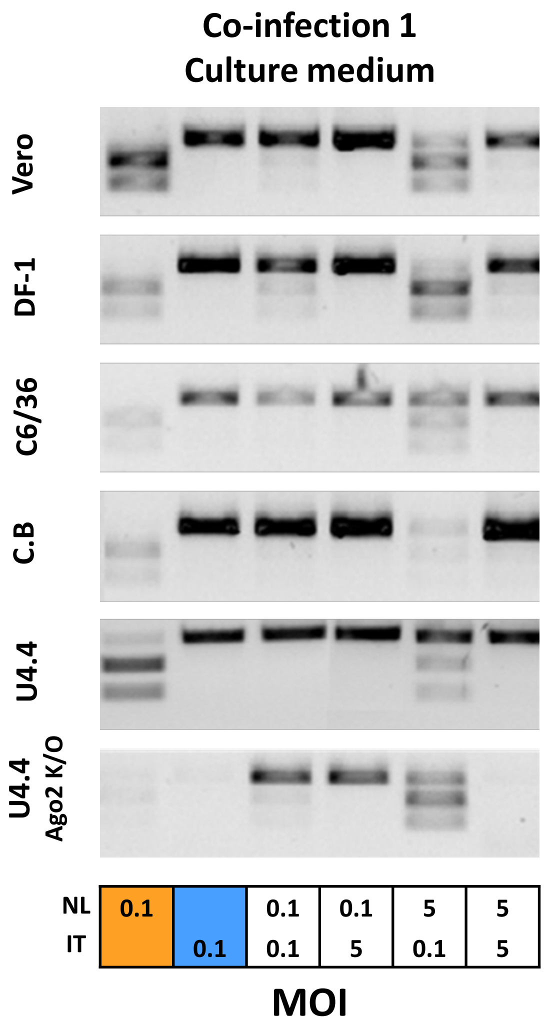

Figure S3. Vero, DF-1, C6/36, C. B, U4.4 WT and U4.4 Ago2 k/o cells were inoculated with both USUV isolates at different MOI combinations of either 0.1, 1 or 5. At 3 dpi, cell culture medium were subjected to RNA extraction followed by RT-PCR and restriction enzyme digestion to determine the presence of both USUV isolates.

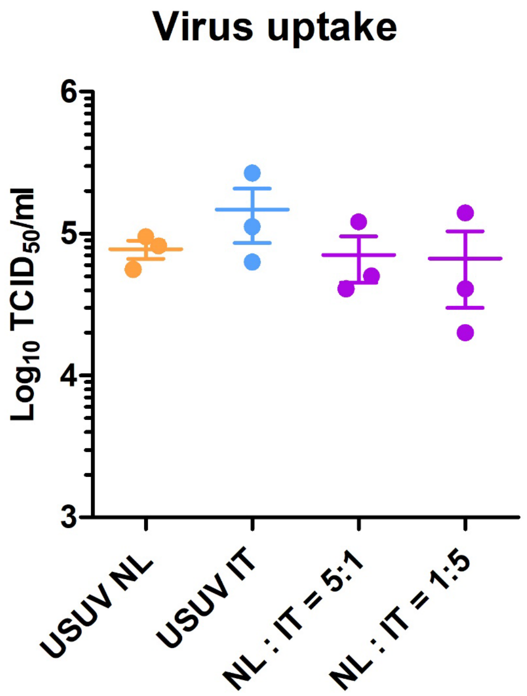

Figure S4. Virus uptake after an USUV-NL and USUV-IT co-infectious bloodmeal in *Culex pipiens* mosquitoes. Virus uptake was checked immediately after the blood meal.
